# Three Journal Similarity Metrics and Their Application to Biomedical Journals

**DOI:** 10.1371/journal.pone.0115681

**Published:** 2014-12-23

**Authors:** Jennifer L. D′Souza, Neil R. Smalheiser

**Affiliations:** 1 Department of Computer Science, University of Texas at Dallas, Richardson, Texas, United States of America; 2 Department of Psychiatry, University of Illinois at Chicago, Chicago, Illinois, United States of America; University of Memphis, United States of America

## Abstract

In the present paper, we have created several novel journal similarity metrics. The MeSH odds ratio measures the topical similarity of any pair of journals, based on the major MeSH headings assigned to articles in MEDLINE. The second metric employed the 2009 Author-ity author name disambiguation dataset as a gold standard for estimating the author odds ratio. This gives a straightforward, intuitive answer to the question: Given two articles in PubMed that share the same author name (lastname, first initial), how does knowing only the identity of the journals (in which the articles were published) predict the relative likelihood that they are written by the same person vs. different persons? The article pair odds ratio detects the tendency of authors to publish repeatedly in the same journal, as well as in specific pairs of journals. The metrics can be applied not only to estimate the similarity of a pair of journals, but to provide novel profiles of individual journals as well. For example, for each journal, one can define the MeSH cloud as the number of other journals that are topically more similar to it than expected by chance, and the author cloud as the number of other journals that share more authors than expected by chance. These metrics for journal pairs and individual journals have been provided in the form of public datasets that can be readily studied and utilized by others.

## Introduction

Scientific journals are major entities studied in bibliometrics and scientometrics. Profiling journals, and measuring how different journals relate to each other, are only two of the aspects that have been examined. For example, journals can be characterized and related according to their citation patterns [1]–[2], topical similarity [3], keyword similarity [4], user behavior during PubMed weblog sessions [5] and even the degree of interlocking editorships [6]. Cordier [7] proposed relating journals according to the number of authors that they share, though this idea has not yet been implemented nor tested.

The Author-ity author name disambiguation 2009 dataset [8] consists of all articles that have PubMed identifiers, published 1950–July 2009, with at least one listed author. For each author name listed on any article, the dataset assigns the articles bearing that name to a set of predicted distinct author-individuals. Author-ity is based on a pairwise machine learning model [9] that examines two articles that share the same (last name, first initial) in the author field, and predicts the probability that the two names refer to the same individual. The model employs multiple features including both author features (e.g., matches on first name, middle initial and suffix) and article features (e.g., matches on title words, affiliations, journal names, emails, etc.).

The current Author-ity model scores journal match in all-or-none fashion. If two articles are published in the same journal, the feature score  = 1, and otherwise  = 0. However, this scoring scheme does not take into account partial similarity between journals. Journals can be similar topically (e.g. *Neurology* vs. *Annals of Neurology*), or due to author publication behavior, that is, the fact that individuals are likely to publish in a constrained range of journals. We have investigated this question in the present study, and have asked: Given any two articles in PubMed that share the same author name (lastname, first initial), how does knowing only the identity of the journals (in which the articles were published) predict the relative likelihood that they are written by the same author-individual? Three different pairwise journal metrics were computed and characterized, which capture different aspects of journal relationships, and directly or indirectly reflect author publication behavior. These studies provide new features to augment and correct erroneous assignments within the Author-ity disambiguation effort. More generally, the author-based metrics are novel and may have wider usefulness for studies of journals, disciplines and scientometrics.

## Methods

The 2009 Author-ity dataset is based on a snapshot of PubMed (which includes both MEDLINE and PubMed-not-MEDLINE records) taken in July 2009, including a total of 19,011,985 Article records, 61,658,514 author name instances and 20,074 unique journal names. Each instance of an author name is uniquely represented by the PMID and the position on the paper (e.g., 10786286_3 is the third author name on PMID 10786286). Thus, each predicted author-individual is associated with a list of predicted PMIDs written by that individual.

For all metrics, we filtered the set of journals to retain only the 9,284 journals that have at least 100 articles within the Author-ity dataset, in order to ensure statistical robustness and to exclude journals that are very old or very new. This generated 13,129,909 pairs of journals which had at least one predicted author-individual in common. To characterize the author metric, this set was filtered further to retain only those journals which have at least 10 distinct authors in Author-ity, giving 9,281 unique journals and 13,129,781 journal pairs. To characterize the MeSH metric, the original set was filtered to retain only journals having at least 50 major MeSH terms within MEDLINE (1950–2012), giving 8,900 journals and 12,569,613 journal pairs. Finally, to compare the metrics, we created a single filtered set of journals, having at least 100 articles and 10 authors within Author-ity, and at least 50 major MeSH terms in MEDLINE, giving 8,897 journals and 12,569,485 journal pairs.

### MeSH-based metric

**Figure 1:**
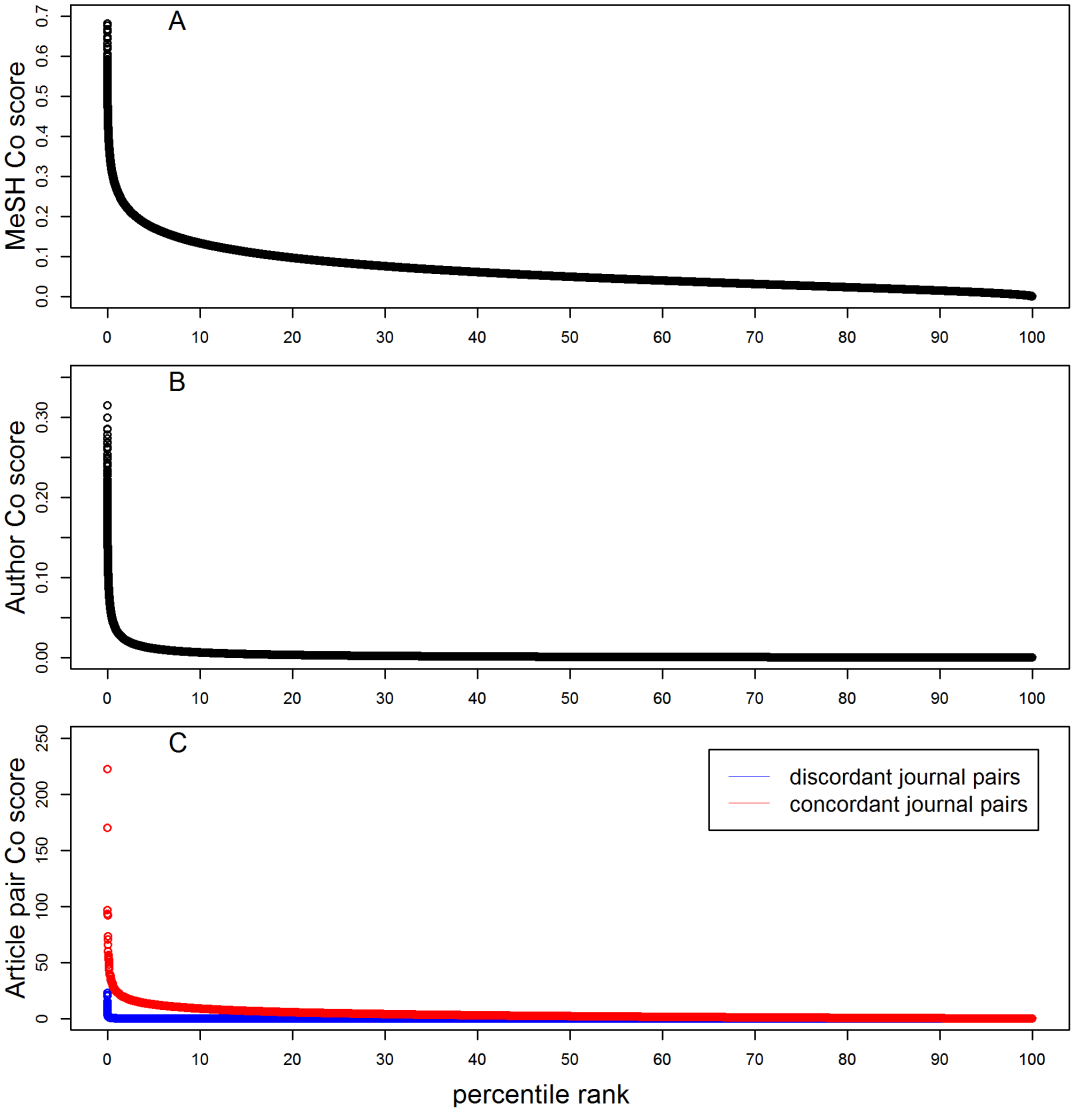
Journal pairs ranked by their normalized co-occurrence (Co) scores. A random sample of 500,000 journal pairs was taken (from a total of 12,569,485 pairs in the study dataset) and plotted to show (A) MeSH Co score, (B) Author Co score, and (C) article pair Co score. All concordant journal pairs have MeSH and author Co scores  = 1, and are not displayed in panels A and B. In panel C, article pair Co scores for concordant and discordant journal pairs are shown separately.

For each journal listed in the 2009 Author-ity dataset we identified all PMIDs of articles published in that journal that were included in MEDLINE, and made a list of all major Medical Subject Headings (MeSH) associated with the articles. To compare two journals pairwise with regard to MeSH term similarity, we scored the number of MeSH terms in common between the two journals, giving larger weight to terms that occurred in multiple articles. For example, if a given MeSH term was assigned to 3 articles in Journal 1 (J1) and 10 articles in Journal 2 (J2), we would score this term as having weight 3. The final MeSH normalized co-occurrence (Co) score is the weighted sum over all MeSH terms, normalized by journal size (geometric mean of the total number of unique MeSH terms assigned to articles in each journal) (Fig. 1A). In order to ensure that the metric would be robust and meaningful, comparisons were only made for journals that had at least 50 major MeSH headings.
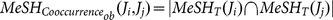
(1)

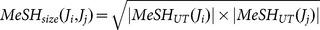
(2)

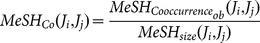
(3)where, 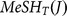 are all major MeSH terms in journal *J*, and 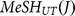 are the unique major MeSH terms in journal *J*.

**Figure 2:**
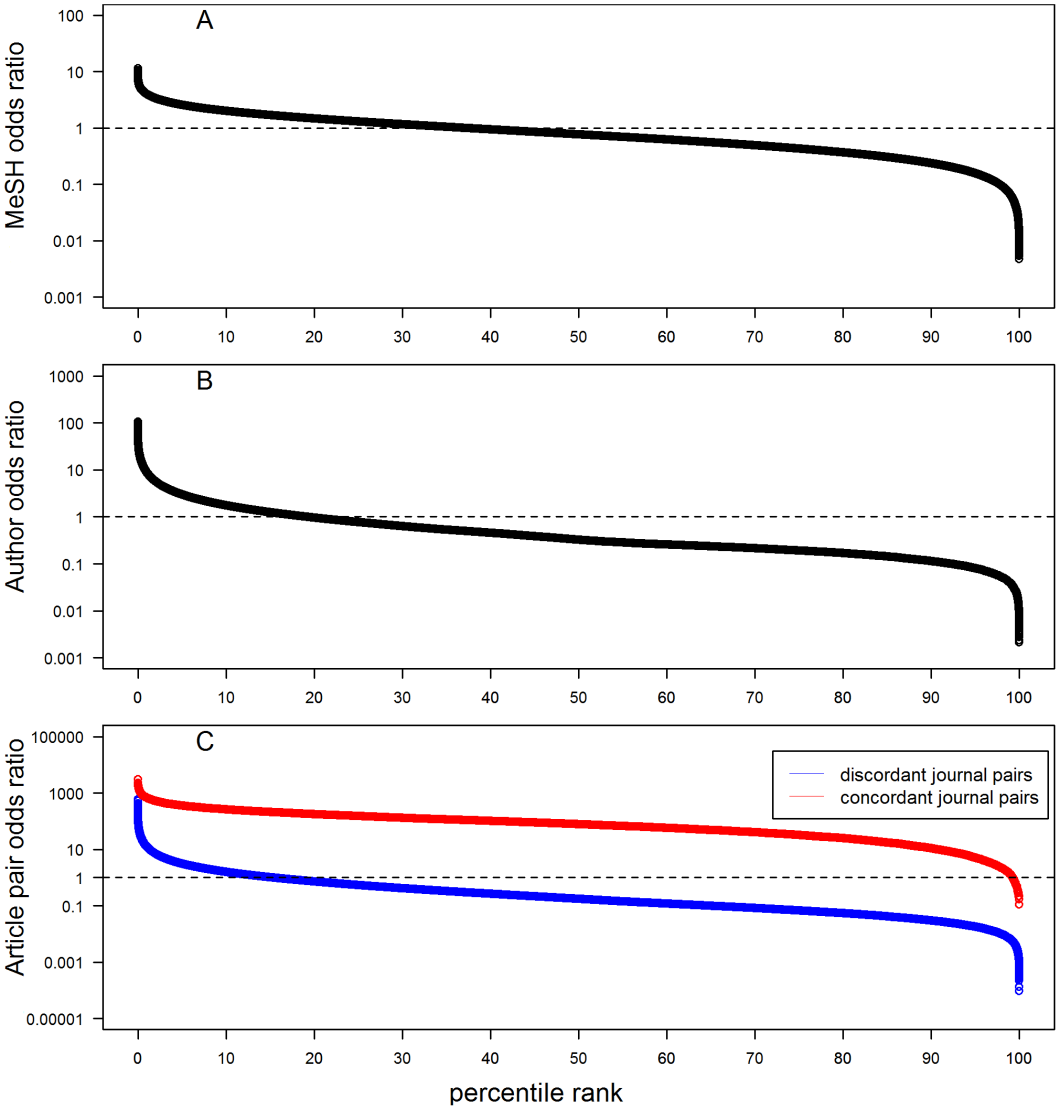
Journal pairs ranked by their odds ratios. A random sample of 500,000 discordant journal pairs was taken (from a total of 12,569,485 pairs in the study dataset) and plotted to show (A) MeSH odds ratio, (B) Author odds ratio, and (C) article pair odds ratio. In addition, concordant journal pairs are shown for the article pair odds ratio. Note that the odds ratios are displayed on a log scale. A dotted line indicates the point at which the odds ratio  = 1; points above this line show more observed co-occurrences than expected by chance, whereas those below the line show fewer than expected by chance. See Methods for details.

Next, we computed the Co score that would be expected simply by chance (for two journals of their size). This was done by ranking all journal pairs by journal size, dividing into bins of 5,000 pairs (each having roughly the same journal size), and calculating the average Co score across all journal pairs in the same bin. Finally, we calculated the MeSH odds ratio for each pair of journals present in that bin, by dividing the observed Co score by the Co score expected by chance (Fig. 2A).
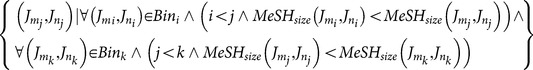
(4)

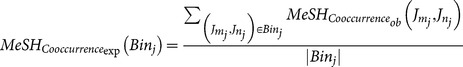
(5)where, 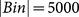 and 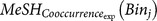 is the major MeSH term co-occurrence expected by chance for all 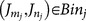.
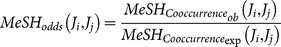
(6)


### Author-based metric

Using the Author-ity 2009 dataset as a gold standard, we scored the author Co score as the number of predicted author-individuals in common between each pair of journals, normalized by journal size (geometric mean of the total number of unique author-individuals publishing in each journal) (Fig. 1B).
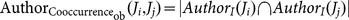
(7)

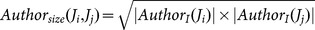
(8)

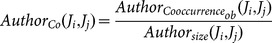
(9)where, 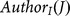 are the unique author-individuals publishing in journal 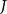.

Next, we computed the author Co score that would be expected simply by chance (for two journals of their size). This was done by dividing all journal pairs into bins of 5,000 pairs, each having roughly the same journal size, and calculating the average number of author-individual co-occurrences across all journal pairs in the same bin. Finally, we calculated the author odds ratio for each pair of journals, by dividing the observed Co score by the Co score expected by chance (Fig. 2B). In order to ensure that the metric would be robust and meaningful, comparisons were only made for journals that had at least a total of 10 or more predicted author-individuals.
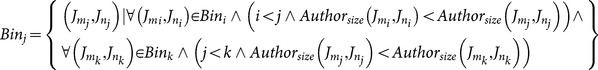
(10)

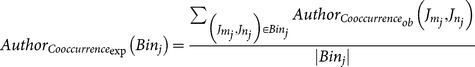
(11)where, 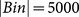 and 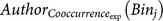 is the author co-occurrence expected by chance for all 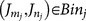.
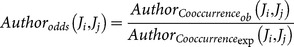
(12)


### Article pair based metric

Using the Author-ity 2009 dataset, we compiled all article pairs that co-occur within each author-individual cluster, i.e., that are predicted to be written by the same author-individual, and compiled a list of all such article pairs across all individuals. After mapping the PMIDs of these co-occurring article pairs to their journals, we obtained 13,129,909 unique journal pairs – including pairs in which both articles were published in the same journal, which allowed us to assess the relative tendency of individuals to publish repeatedly in the same journal over time. The article pair Co score equals the total number of co-occurrences for that journal pair divided by the geometric mean of the journal sizes (i.e., total number of articles that map to each journal) (Fig. 1C).
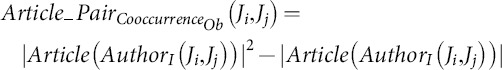
(13)

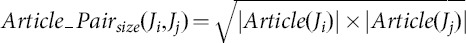
(14)

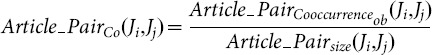
(15)where, 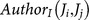 is the set of author-individuals who have published articles in both journals 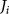 and 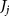, and 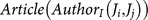 is the list of all articles from either 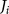 or 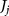 by authors that the two journals share in common, and 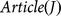 is all the articles in journal 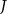. In the mathematical formula for observed article pair co-occurrence, 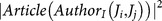 gives the total number of article pair combinations from the two journals where the articles are authored by author-individuals the two journals share in common. Since the count also includes an article paired with itself, subtracting the total number of the articles, i.e., 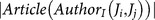 removes the added effect.

Next, we computed the article pair Co score that would be expected simply by chance (for two journals of their size). This was done by dividing all journal pairs into bins of 5,000 pairs, each having roughly the same journal size, and calculating the average Co score across all journal pairs in the same bin. Finally, we calculated the article pair odds ratio for each pair of journals, by dividing the observed Co score by the Co score expected by chance (Fig. 2C). In order to ensure that the metric would be robust and meaningful, comparisons were only made for journals that had either 10 or more observed article pair co-occurrences, or 10 or more co-occurrences expected by chance.
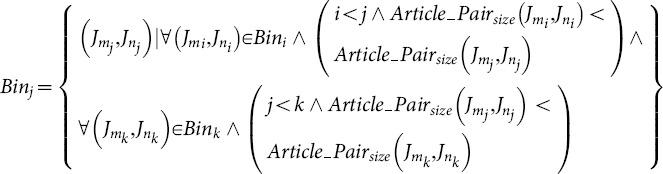
(16)

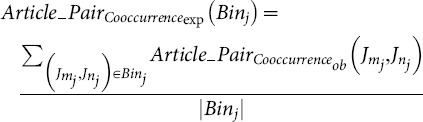
(17)where 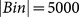 and 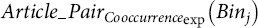 is the article pair co-occurrence expected by chance for all 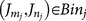.
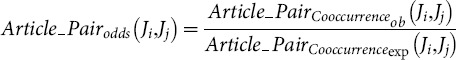
(18)


### Disciplines

The 2011 release of the Text Categorization (TC) toolkit developed at NLM (http://lexsrv3.nlm.nih.gov/LexSysGroup/Projects/tc/2011/web/index.html) was used to annotate journals with their disciplines. For most journals, pre-computed journal descriptor annotations were available; 300 journals were not included, and for these we computed annotations directly from the JDI (Journal Descriptor Indexing) tool included in the TC package. The tool takes as input a journal's MeSH terms (both major and non-major terms were included), and based on a similarity measure, provides a ranked list of disciplines from most to least similar. We used the highest ranked discipline as the journal's discipline (though the pre-computed annotations sometimes consisted of two or three categories simultaneously). These discipline categories, which are thus MeSH terms, are selected from a pre-created list of 122 unique categories [3]. For the most part, the JDI categories were similar to those assigned in the NLM catalog and corresponded to common sense. However, JDI had limited coverage in non-biomedical fields. For example, *Astrophysical Journal* was indexed as Chemistry, Analytical, *Physical Review Letters* was indexed as Medicine, and *Los Alamos Science* as Environmental Health. We manually changed the latter two to Physics, which otherwise does not exist as a JDI category. Because of these and other errors, JDI discipline categories were simply used for display purposes and were not incorporated into any metrics. Nevertheless, we preferred use of JDI instead of NLM categories (which did not cover all journals in Author-ity) or Web of Science categories (which are manually assigned rather than computed according to a standard terminology and reproducible algorithm [10]).

### Statistics

We employed correlation measures to characterize the relationship between two metrics, which allowed us to estimate the similarity of the metrics. We also employed correlation measures between a metric and a potential contributing factor (e.g. age of a journal), to estimate how much of the variation in metric scores can be attributed to variation in the contributing factor (or in other factors linked to the contributing factor). In general, the nonparametric Spearman rho rank correlation coefficient is more appropriate for these comparisons, because the metrics and factors are generally not linear. However, we also present the parametric Pearson r correlation coefficient as well, since there is some value in comparing the Pearson and Spearman values (e.g., if both are high, the relationships are likely to be linear, whereas if Pearson is very low and Spearman is very high, the relationships are likely to be nonlinear). Because each correlation was computed across millions of datapoints, statistical significance is generally extremely high and p-values are not displayed.

## Results

### A. Metrics for relating similarity of journals considered pairwise

#### 1. MeSH-based metric

The MeSH normalized co-occurrence (Co) score (Fig. 1A) provides a measure of the intrinsic topical similarity between two journals J1 and J2, ranging from near 1 down to 0.00041 for the most dissimilar journal pairs. This metric is straightforward to interpret, and is derived from curated MEDLINE data which is independent of Author-ity. The metric can be used to generalize the existing Author-ity pairwise model by marking journal pairs not simply as nonmatch  = 0 or match  = 1, but varying between 0 and 1 according to their Co score. However, because the Co score is influenced by journal size, perhaps a more useful way to employ the MeSH metric is to examine the odds ratio, which calculates the extent to which the observed Co score is greater or less than expected by chance (Fig. 2A). The MeSH odds ratio scales the journal similarity to fit directly into the Author-ity pairwise model (each of whose feature scores should ideally represent odds ratios [9]). About 37% of journal pairs have MeSH odds ratios above 1, ranging down to a minimum value at about 0.0016.

#### 2. Author-based metric

The 2009 Author-ity disambiguation dataset [7] was used as a gold standard to score journal pairs according to how many distinct author-individuals have published in both journals. The author Co score is shown in Fig. 1B, but again, the most relevant parameter for disambiguation is the author odds ratio, which is plotted in Fig. 2B. The maximal odds ratio is 139, for *Japanese Journal of Thoracic and Cardiovascular Surgery* paired with *Annals of Thoracic Cardiovascular Surgery*. An odds ratio of 139 means that (holding all other factors constant) if two articles sharing the same author name are written in these two journals, then the chances that the two articles were written by the same individual are 139 times greater than the chance that they were written by different individuals. Only about 19% of journal pairs have odds ratio at or above 1, however. The minimum odds ratio is 0.0018, for *Soviet Medicine* paired with *Applied Environmental Microbiology*; this means that (holding all other factors constant) the chances that two articles sharing the same author name (written in these two journals) are written by the same individual are only 0.0018 times the chance that they were written by different individuals. Thus, the author odds ratio provides an estimate of the effect of journal identity, which is based on the tendency of individuals to publish within a constrained range of journals. Because the data are computed across all authors in PubMed, and because we excluded journals that have less than 100 articles or 10 authors in Author-ity, the data are likely to be robust.

Note that the Author-ity dataset contains ∼1–2% of erroneous article assignments overall, comprising lumping errors (articles from two different individuals are placed in the same author-individual cluster) and splitting errors (articles from one individual are placed in two or more clusters) [8]. Errors are greatest for extremely common author names such as J. Lee [8], [9]. Errors in which different author-individuals are counted as the same person would cause some journal pairs to exhibit inflated odds ratios; conversely, errors in which the same individual is counted as distinct authors would deflate the odds ratio estimate. To assess the impact of errors in the gold standard, we simulated a near worst-case scenario in which two journals J1 and J2 are both very small (each have only 100 articles in Author-ity) and predicted to share 10 author-individuals, yet in reality one of those matches is an error and they truly share only 9 author-individuals. This produces an error of 11%. Most journal pairs in our dataset that have author odds ratio estimates above 1 are much larger than this and share many more authors, so the potential errors are likely to be much smaller in most cases. (Note that even extremely large percent errors in odds ratios may have little consequence for disambiguation in the majority of cases: When the odds ratio is far below 1, which is true for the majority of journal pairs, whether the true odds ratio is 0.005, 0.1 or 0.15 does not matter in practical terms. Similarly, for odds ratios well above 1, the exact value (e.g., 20 vs. 40) is of little consequence.)

The author odds ratio complements the topical information given by the MeSH metric. Two journals may cover the same topic, and yet rarely contain articles from the same author. For example, the Japanese journal *Jinko Mondai Kenkyu* (translated name: *Journal of Population Problems*) and *European Journal of Population* are both concerned with population studies (MeSH odds ratio  = 10.5) but have few predicted authors in common (author odds ratio  = 0.081).

#### 3. Article pair metric

The article pair metric introduces a new aspect to the comparison of two journals. If the same author-individual published 3 articles in J1 and 10 articles in J2, then the number of article pairs in J1:J1 is 3 ( = 3 choose 2), the number of pairs in J2:J2 is 45 ( = 10 choose 2) and the number of pairs in J1:J2 is 30 ( = 3*10). These co-occurrence scores are tabulated and added up for all authors, articles and journal pairs in Author-ity (subject to the filtering criteria described in Methods). The article pair odds ratio thus measures the tendency of an author to publish repeatedly, in different journals as well as in the same journal (Fig. 2C).

The maximum article pair odds ratio is surprisingly high (3,051), for the journal *AIDS Treatment News* paired with itself; this reflects the fact that a single person, J.S. James, accounted for 449 articles, or more than half of the total articles published in the entire journal. (Such behavior is not captured by the author metric, which does not give weighting to the number of articles published by an individual in a journal.) As shown in Fig. 2C, the tendency for authors to publish repeatedly in the same journal is, in general, much higher than for discordant journal pairs: the median odds ratio is 73, and 99.2% of same-journal pairs have odds ratios greater than 1, In contrast, only 15% of discordant journal pairs have odds ratios greater than 1.

Some journals are much more likely than others to attract multiple articles from the same author. The range of article pair odds ratios varies widely, from a high of 3,051 (as mentioned above) to a low of 0.11 for *Biochemical Clinics*, a journal that was only published for one year, from 1963 to 1964. The type of article published in the journal appears to be the major factor determining the odds ratio. For example, the journals having the highest article pair odds ratios include newsletters (e.g. *AIDS Treatment News*, *Harvard Women's Health Watch*, etc.) and regional or highly specialized journals (*Archives of the Institute Pasteur of Madagascar*, *Archives of Stomatology (Napoli)*, *Korean Journal of Parasitology*, etc.). These are all dominated by a few authors. Conversely, the journals having the lowest article pair odds ratios tend to be review journals: For example, *The Harvey Lectures* (odds ratio  = 0.31) consists of transcriptions of invited review lectures, rather than author-submitted research articles. Very few individuals are invited to give more than one Harvey Lecture. Other journals with relatively low article pair odds ratios include *Pathobiology Annual*, *Annual Review of Biophysics and Biophysical Chemistry*, *International Review of Physiology*, etc. The large, high-impact multidisciplinary and leading disciplinary journals had high but not extreme values (e.g., *Science* (odds ratio  = 173), *J. Biol. Chem*. ( = 83), *Cell* ( = 39), *Nature* ( = 38), *Proc. Natl Acad. Sci. USA* ( = 37), *J. Neurosci*. ( = 22), *Plos One* ( = 10)).

#### 4. Comparing the three odds ratio estimates

The three metrics gave complementary views of pairs of discordant journals (i.e., cases in which J1 ≠ J2). As expected, the author odds ratio is highly correlated with the article pair odds ratio across discordant journal pairs (r = 0.61, rho = 0.85). (Because of the multiplicative effect of considering article pairs, the article pair odds ratio is relatively sensitive to errors in the existing Author-ity dataset, and so the author metric is preferred as a feature for disambiguation.) And, as expected, the odds ratios are not affected by variations in journal sizes (author vs. geomean of journal size: r = 0.0001, rho = −0.05; article pair vs. journal size, r = −0.001, rho = −0.14; MeSH vs. journal size, r = −0.0002, rho = 0.014). However, the author and article pair odds ratios are only partially correlated with the MeSH odds ratio (author vs. MeSH, r = 0.47, rho = 0.45; article pair vs. MeSH, r = 0.37, rho = 0.375), confirming that author-based journal similarity reflects patterns of publication behavior that only partially reflect topical similarity of the journals.

To illustrate a specific example, Table 1 shows the 20 journals most related to the journal “Bioinformatics” published by Oxford University Press, according to the MeSH vs. author vs. article pair metrics. Overall, the 3 lists include a similar set of journals in bioinformatics, computational biology, and genomics. However, there are some large differences in detail; for example, the highest ranked journal by article pair odds ratio is *Genome Informatics*, whereas it is not in the top 20 by MeSH odds ratio. Conversely, *IEEE Transactions on Image Processing* is ranked #7 by MeSH but is not in the top 20 by either author or article pair rankings. All three metrics gave results that are quite different from the previous Related Journals weblog metric computed by Lu et al. [5], which computes the probability that a user who clicks on one or more articles published in one journal (within a PubMed query retrieval session) will also click on one or more articles published in a second journal during the same session. Over half of the Lu et al.'s top 20 list consists of general and biological journals (Table 1) that are missing from the lists produced by the metrics introduced here.

**Table 1:** The journals most related to the journal *Bioinformatics* according to various metrics.

most related by MeSH	MeSH oddsratio	most related by shared authors	Author oddsratio	most related by article pairs	article pair oddsratio	weblogs
Bioinformatics	9.4	Bioinformatics	118.7	Genome Inform	85.6	BMC Bioinformatics
BMC Bioinformatics	6.9	Brief. Bioinformatics	54.5	J. Comput. Biol.	78.6	*Nucleic Acids Res.*
J. Comput. Biol.	4.3	J. Comput. Biol.	53.9	Proc Int Conf Intell Syst Mol Biol	73.3	*Proc. Natl. Acad. Sci. U.S.A.*
PLoS Comput. Biol.	3.7	Proc Int Conf Intell Syst Mol Biol	50.0	Pac Symp Biocomput	69.1	Genome Res.
Pac Symp Biocomput	3.6	BMC Bioinformatics	47.7	BMC Bioinformatics	66.9	*Nature*
Comput. Appl. Biosci.	3.6	Pac Symp Biocomput	47.5	Brief. Bioinformatics	58.8	*Science*
IEEE Trans Image Process	3.5	J Bioinform Comput Biol	43.2	Genome Res.	53.6	*J. Mol. Biol.*
Genome Biol.	3.4	PLoS Comput. Biol.	38.2	J Bioinform Comput Biol	52.0	Proteins
J Bioinform Comput Biol	3.4	IEEE/ACM Trans Comput Biol Bioinform	34.9	Bioinformatics	52.0	*J. Biol. Chem.*
IEEE/ACM Trans Comput Biol Bioinform	3.3	Genome Inform	33.9	PLoS Comput. Biol.	45.7	Genome Biol.
Brief. Bioinformatics	3.2	In Silico Biol. (Gedrukt)	31.9	Genome Biol.	44.3	BMC Genomics
Genome Res.	3.1	BMC Syst Biol	30.6	Mol. Syst. Biol.	40.2	*Methods Mol. Biol.*
IEEE Trans Pattern Anal Mach Intell	3.1	Genome Biol.	30.4	Comput. Appl. Biosci.	39.2	*Nat. Genet.*
BMC Genomics	3.1	Stat Appl Genet Mol Biol	28.6	BMC Syst Biol	35.6	PLoS Comput. Biol.
BMC Syst Biol	3.0	Comput. Appl. Biosci.	27.8	In Silico Biol. (Gedrukt)	35.4	*Cell*
IEEE Trans Syst Man Cybern B Cybern	2.9	Appl. Bioinformatics	26.9	Curr Protoc Bioinformatics	34.9	J. Comput. Biol.
Med Image Comput Comput Assist Interv	2.9	Mol. Syst. Biol.	26.6	IEEE/ACM Trans Comput Biol Bioinform	34.0	*Nat. Biotechnol.*
IEEE Trans Med Imaging	2.9	Comput Biol Chem	25.8	OMICS	32.9	*Mol. Biol. Evol.*
BioSystems	2.8	OMICS	23.3	Stat Appl Genet Mol Biol	32.0	*PLoS ONE*
Proc Int Conf Intell Syst Mol Biol	2.8	Curr Protoc Bioinformatics	21.1	Proteins	31.1	Pac Symp Biocomput

The 20 journals most related to the journal “Bioinformatics” are displayed according to MeSH odds ratio, author odds ratio, and article pair odds ratio. For comparison, the 20 most related journals are displayed using the metric of Lu et al. [5] which is based on user clicking behavior during weblogs of PubMed retrieval sessions. Note that the first three metrics measures the similarity of “Bioinformatics” to itself, though Lu et al. do not. Note, also, that over half of the top journals listed by the weblog metric are biological and general journals (shown in italics) which are not included in any of the metrics reported in the present paper.

### B. What do these journal metrics tell us about journals, and how they are related to each other?

Journals can be characterized and classified in many different ways. The type of articles published may vary (e.g., research articles vs. reviews vs. clinical case reports vs. news items). Journals may have international, regional or even very limited scope (e.g., arising from a single research institute). Topically, journals may be multidisciplinary, disciplinary or highly sub-specialized. Within a given field or scientific community, different journals may vary in size, popularity, prestige, and impact factor, and a journal's status may be central, marginal or even negative (i.e., a journal that is widely viewed as not reputable).

The simplest possible journal measure is simply to rank journals in terms of their size. (Size can be defined in terms of the number of articles, number of authors or number of MeSH terms; all three measures are very highly correlated and give essentially similar results. Note, however, that we have defined journal size in this section as the number of articles included within the 2009 Author-ity dataset.). As shown in Table 2, the 25 largest journals consist mostly of multidisciplinary and leading disciplinary journals.

**Table 2:** Top 25 journals sorted by journal size (as of 2009).

	Journal	Size	Discipline
1	*J. Biol. Chem.*	147822	Biochemistry
2	*Science*	134066	Science
3	*Lancet*	102954	Medicine
4	*Proc. Natl. Acad. Sci. U.S.A.*	96559	Molecular Biology
5	*Biochim. Biophys. Acta*	85035	Biochemistry; Biophysics
6	*Nature*	83552	Science
7	*Biochem. Biophys. Res. Commun.*	67414	Biochemistry; Biophysics
8	*Biochemistry*	56342	Biochemistry
9	*N. Engl. J. Med.*	55918	Medicine
10	*Am. J. Physiol.*	54202	Physiology
11	*J. Immunol.*	53305	Allergy and Immunology
12	*JAMA*	53274	Medicine
13	*Brain Res.*	51790	Brain
14	*Biochem. J.*	51371	Biochemistry
15	*J. Bacteriol.*	46506	Bacteriology
16	*BMJ*	44459	Medicine
17	*Cancer Res.*	44057	Neoplasms
18	*Ann. N. Y. Acad. Sci.*	43766	Science
19	*FEBS Lett.*	40385	Biochemistry
20	*J. Urol.*	38813	Urology
21	*Ugeskr. Laeg.*	36200	Orthopedics
22	*Nippon Rinsho*	34920	Medicine
23	*J. Physiol. (Lond.)*	34880	Physiology
24	*Cancer*	34531	Neoplasms
25	*Br Med J*	34472	Medicine

Shown are the ISO-abbreviated journal names. Journal size is defined as number of articles included within the 2009 Author-ity dataset. Note that some distinct journal names (e.g. BMJ and Br Med J) are successors of each other in different time periods. Disciplines are taken from JDI annotations. See Methods for details.

Another simple measure is to divide the total number of unique major MeSH terms assigned to articles published in a journal, by the total number of their articles that are indexed by MeSH terms, to create an indicator of topical broadness. Unfortunately, this broadness measure does not scale well with journal size; it tends to overestimate the broadness of small journals and to underestimate the broadness of large journals (data not shown). Instead, a different, novel and potentially interesting measure is to employ the MeSH-based odds ratio. For each journal, one can envision that there is a “cloud” of other journals which are topically related to it more than expected by chance. That is, for each journal J1 one can count the number of journals Jx for which the MeSH odds ratio for the journal pair J1:Jx is greater than 1. All other factors being equal, journals of larger size (i.e., having larger numbers of MeSH terms) will tend to have a larger MeSH cloud, but these measures are only partially correlated (Pearson r = 0.29, Spearman rho = 0.63). Journals having higher broadness indices actually have a slight tendency to have smaller MeSH clouds (r = −0.16), indicating that the broadness index and the MeSH cloud are quite different measures (see also Tables 3 and 4). The 25 journals with the largest MeSH clouds are mostly clinical or investigative medical journals and include some general journals as well, e.g., *Medical Hypotheses* and *Scientific American* (Table 3).

**Table 3:** Top 25 journals sorted by size of MeSH cloud.

	Journal	Discipline	Broadness index	MeSH cloud
1	*Med. Sci. Monit.*	Medicine	1.25	5399
2	*Isr. J. Med. Sci.*	Medicine	0.88	5332
3	*Med. Hypotheses*	Medicine	0.60	4885
4	*Arthritis Rheum.*	Rheumatology	0.58	4830
5	*Postgrad Med J*	Medicine	0.82	4784
6	*Mayo Clin. Proc.*	Medicine	0.96	4689
7	*Br. Med. Bull.*	Medicine	1.22	4555
8	*Prog. Clin. Biol. Res.*	Biology; Medicine	0.46	4548
9	*Ann. Rheum. Dis.*	Rheumatology	0.67	4543
10	*Am. J. Med.*	Medicine	0.59	4512
11	*J. Neurol. Sci.*	Neurology	0.68	4501
12	*Yale J Biol Med*	Biology; Medicine	1.31	4445
13	*Arch. Intern. Med.*	Internal Medicine	0.58	4383
14	*Arch. Pathol. Lab. Med.*	Pathology	0.77	4380
15	*Ciba Found. Symp.*	Medicine	0.92	4355
16	*J. Intern. Med.*	Internal Medicine	0.90	4307
17	*Sci. Am.*	Science	0.94	4249
18	*J. Int. Med. Res.*	Medicine	1.29	4239
19	*Clin Invest Med*	Medicine	1.25	4232
20	*Ann. Acad. Med. Singap.*	Dentistry	1.05	4226
21	*Braz. J. Med. Biol. Res.*	Biology; Medicine	0.99	4218
22	*South. Med. J.*	Medicine	0.60	4213
23	*Acta Neurol. Scand.*	Neurology	0.79	4187
24	*Clin. Pharmacol. Ther.*	Drug Therapy; Pharmacology	0.76	4184
25	*Bull. Acad. Natl. Med.*	Medicine	0.97	4182

**Table 4:** Top 25 journals sorted by author cloud/MeSH cloud ratio.

	Journal	Discipline	Broadness index	MeSH cloud	Size	# authors	author cloud	Author/MeSH cloud
1	*Pharmatherapeutica*	Drug Therapy	1.67	60	321	916	535	8.92
2	*Phytother Res*	Complementary Therapies	0.54	96	2408	8356	842	8.77
3	*Fitoterapia*	Complementary Therapies	0.56	50	1480	4723	432	8.64
4	*Phytomedicine*	Complementary Therapies	0.62	92	1227	4709	763	8.29
5	*J. Biol. Chem.*	Biochemistry	0.10	151	147822	248546	1145	7.58
6	*Monogr Hum Genet*	Genetics	1.52	82	143	365	612	7.46
7	*J Ethnopharmacol*	Pharmacology; Social Sciences	0.44	105	5221	14841	760	7.24
8	*CNS Drug Rev*	Neurology; Pharmacology	1.90	73	153	486	404	5.53
9	*Skin Pharmacol.*	Dermatology; Pharmacology	0.94	136	360	1000	713	5.24
10	*Sarcoidosis*	Allergy and Immunology	0.97	146	411	907	758	5.19
11	*Cancer Genet. Cytogenet.*	Genetics, Medical; Neoplasms	0.13	324	6592	17288	1640	5.06
12	*Adv Biosci*	Biology; Reproductive Medicine	1.23	135	216	517	646	4.79
13	*Expert Opin Emerg Drugs*	Drug Therapy	0.98	260	321	739	1236	4.75
14	*Jpn J Antibiot*	Anti-Bacterial Agents	0.45	168	5107	10268	776	4.62
15	*Biomed. Environ. Mass Spectrom.*	Biochemistry; Chemistry Techniques, Analytical	1.57	150	526	1465	680	4.53
16	*Biochim. Biophys. Acta*	Biochemistry; Biophysics	0.18	380	85035	132654	1716	4.52
17	*Adv Prostaglandin Thromboxane Res*	Biochemistry	0.89	225	496	1173	986	4.38
18	*Planta Med.*	Botany	0.46	175	6137	14978	766	4.38
19	*J Pharmacol*	Pharmacology	1.34	202	342	794	837	4.14
20	*Nat. Prod. Res.*	Biology	1.26	84	1028	3287	347	4.13
21	*Hum. Genet.*	Genetics, Medical	0.19	546	9053	25992	2211	4.05
22	*Nature*	Science	0.18	448	83552	138529	1792	4.00
23	*Ther Apher*	Hematology	0.60	200	420	1260	798	3.99
24	*Eur J Toxicol*	Toxicology	1.62	73	132	303	275	3.77
25	*Zhongguo Zhen Jiu*	Complementary Therapies	0.33	51	730	2051	192	3.76

In this Table, journal size is defined as number of articles with listed authors in the Author-ity (2009) dataset.

In this Table, journal size is defined as number of articles with listed authors in the Author-ity (2009) dataset.Alternatively, journals can be characterized according to the size of their author cloud. That is, for each journal J1 one can count the number of journals Jx for which the author odds ratio for the journal pair J1:Jx is greater than 1. This measures the tendency of authors who publish in J1 to publish in other specific journals Jx as well. All other factors being equal, journals of larger size will tend to have a larger author cloud (Pearson r = 0.39, Spearman rho = 0.62). Although the size of the MeSH cloud and author cloud are rather highly correlated over the entire dataset (r = 0.78, rho = 0.82), certain journals have a much larger or smaller author cloud than expected for the size of its MeSH cloud. As shown in Table 4, the 25 journals with the highest author/MeSH cloud ratios include *Nature*, major biochemistry and pharmacology journals, and several journals devoted to drugs derived from plants or natural products. High author/MeSH cloud ratios indicate that these journals draw authors from relatively many arenas. The journals with the lowest author/MeSH cloud ratios are quite different in profile, comprising general magazines in which the authors are likely to be journalists (e.g., *Time*, *Wall St. Journal*, *Fortune*), professional magazines and newsletters (e.g., *Kentucky Hospitals Magazine*, *State Legislatures*, *Executive Housekeeping Today*, *FDA Consumer*), and some small nursing journals. Low author/MeSH cloud ratios indicate that the journal draws from only a small community of authors, much smaller than the potential pool that might potentially write on related topics.

### C. Evaluation of the author odds ratio for error correction of the 2009 Author-ity dataset

Within the Author-ity dataset, there are many “singleton” author-individual clusters, consisting of single articles that cannot be assigned to any other cluster with high confidence. In some cases this may be a correct assignment (i.e., that author may have published only a single article that is indexed in MEDLINE), but often, this reflects a lack of available information to be provided to the existing pairwise disambiguation model. We carried out a preliminary study to assess whether the author odds ratio could provide new information that would allow correct assignments of individual articles.

First, we characterized the distribution of average author odds ratios for “singleton” cluster articles compared to the three largest clusters having at least 10 articles per cluster and bearing the same author (last name, first initial). This set, comprising a mixture of singletons that are appropriately and inappropriately assigned, has author odds ratio mean  = 1.5, SD = 3.49, and an upper 95% confidence value of 8.35. As a negative control, the average author odds ratios were calculated for “singleton” cluster articles compared to the three largest clusters having at least 10 articles per cluster and bearing the same author (last name, first initial), but in which first names were given and mis-matched between singleton and candidate large cluster. The average author odds ratio across the negative control set has mean  = 1.16, SD = 2.58, and an upper 95% confidence value of 6.21.

We then examined all singleton clusters which had average author odds ratios>8.35 for one of the three largest clusters having≥10 articles and sharing the same author (last name, first initial), yet ratios <1 for the other two largest clusters. We manually assessed the 20 singletons which exhibited the highest average author odds ratios (ranging from 38 to 54.4) by examination of the MEDLINE records and full-text of articles, when available. Of thirteen articles that could be identified with high confidence, twelve singletons referred to the same person associated with the large cluster (for example, Robert Usher Newton, School of Exercise, Edith Cowan University, 100 Joondalup Drive, Joondalup, WA, 6027, Australia, writing about sports medicine). Only one of the thirteen was definitely different (a case in which a father and son having the same name published together as co-authors). Of the seven articles that could not be definitely assigned, six were probably referring to the person associated with the large cluster as well, based on multiple similarities of middle name or initial, topic, and city or country of affiliation. Further evaluations are needed to learn how strongly the overall accuracy of the Author-ity dataset will be affected by adding author odds ratios as a new feature in the pairwise disambiguation model. However, these data suggest that adding author odds ratio information should be helpful, particularly for assigning “singleton” clusters that currently remain unassigned to larger clusters.

## Discussion

In the present paper, several novel journal metrics have been created which are related directly or indirectly to author publication behavior. The author odds ratio counts the number of predicted author-individuals publishing in a pair of journals J1 and J2, and divides by the number expected by chance. This is a straightforward, intuitive measure of how authors publish in a constrained range of journals. However, it is subject to several minor limitations. Since it is calculated from the 2009 Author-ity dataset, the odds ratio estimates may be inaccurate by up to 11% in some cases. Also, the odds ratio estimate is not robust for journals that have very few authors publishing in them, or for very new journals.

The MeSH odds ratio measures the topical similarity of any pair of journals, and has the advantage that it is calculated simply from the major MeSH headings assigned to articles (hence it is not affected by errors in the Author-ity dataset). The MeSH odds ratio estimate has several limitations: It is subject to some noise and variation, since MeSH indexing is performed manually, and since indexing terms exhibit some changes over the years. Because MeSH terms are intended to capture biomedical concepts, they may not fully measure non-biomedical papers (e.g. astrophysics, materials science, or arts), and may under-count the true topical diversity of multidisciplinary journals. Finally, the MeSH odds ratio estimate is not robust for journals that have few articles indexed with MeSH terms.

The article pair odds ratio, like the author odds ratio, measures publication behavior of authors, but it specifically asks: Given TWO articles written by the same author-individual, how often will the two be found in a given pair of journals? This metric has the unique advantage that it can detect the tendency of authors to publish repeatedly in the same journal, as well as in different journals. However, due to the multiplicative aspect, it is relatively sensitive to errors in the Author-ity dataset, and so is less preferred for disambiguation purposes. Note that the likelihood of publishing in J2, given that an author has first published in J1, may NOT be the same as the likelihood of publishing in J1 given that he or she has first published in J2. However, our preliminary studies suggest that these two scenarios give similar odds ratios in almost all cases – the exceptions being pairs where one journal is established and the other is very recently launched. To ensure robustness, the article pair odds ratio was only computed for cases in which a threshold number of article pairs (≥10) was observed or expected.

The metrics can be applied not only to estimate similarity of journal pairs, but to provide profiles of individual journals as well. Each journal J1 has a “MeSH cloud” of other journals Jx which are more topically similar to J1 than expected by chance (i.e., have pairwise MeSH odds ratios greater than 1). Similarly, each journal J1 has an “author cloud” of other journals Jx which have pairwise author odds ratios greater than 1 (i.e., in which authors tend to co-publish more than expected by chance). The size and nature of these “clouds” provides a novel perspective for classifying and ranking journals. Journals having a large MeSH cloud, particularly larger than expected for their sizes and for the size of their disciplines, may be cross-disciplinary in scope, whereas journals having a relatively small MeSH cloud may be highly specialized. Journals having a large author cloud (particularly larger than expected for their sizes, the size of their disciplines and the size of their MeSH clouds) seem to attract authors over a wide arc, and in this sense may be said to be “attractive” or “central”. Conversely, journals having relatively small author clouds in relation to their MeSH clouds include “cottage” journals which draw authors from a small community, much smaller than the potential pool that might potentially write on related topics.

We have not attempted to characterize all of the ways in which the journal metrics might be used in scientometrics. For example, they may help in defining disciplines, interdisciplinarity and journal categories in new ways [7]. As well, these journal metrics may be useful (together with other types of information such as citations) for profiling the publication behavior of individual authors. Changes in journal metrics over time may help to track alterations in status of individual journals, and even the evolution of the scientific publishing enterprise. We encourage others to download the datasets and explore their utility.

Our original motivation was to identify different ways of capturing the similarity of two journals, in a manner that will assist us in updating and improving the 2009 Author-ity author name disambiguation dataset [8]. This dataset is already highly accurate, and in fact was utilized as a gold standard here for some purposes; nevertheless, it contains a number of erroneous or uncertain author assignments, and may potentially benefit from including additional journal- related odds ratio estimates in machine learning models. Preliminary studies indicate that the author odds ratio has value in identifying certain articles which share high journal similarity with a large author-individual cluster, and are likely to be written by that individual, yet are assigned to singleton clusters in the current Author-ity dataset. In the future, we plan to test systematically whether one or more of the journal similarity estimates can improve the overall accuracy of author-individual assignments in Author-ity, or help detect which assignments are most likely to be errors.

### Implementation

The datasets and readme.pdf (1.7 MB) are freely available for download from the Arrowsmith project website (http://arrowsmith.psych.uic.edu/arrowsmith_uic/journal_metrics.html) as well as from the UIC Institutional Repository, INDIGO, under the terms of the Creative Commons Attribution-NonCommercial-ShareAlike CC BY-NC-SA license 4.0. The journal pair data is contained in journalPair_metrics.txt (3.96 GB uncompressed, 1.78 GB compressed), and journal data in journal_features.txt (1.08 MB) and journal_features.xlsx (1.14 MB).
